# The Relative Validity of the Menzies Remote Short-Item Dietary Assessment Tool (MRSDAT) in Aboriginal Australian Children Aged 6–36 Months

**DOI:** 10.3390/nu10050590

**Published:** 2018-05-10

**Authors:** Emma Tonkin, Dani Kennedy, Rebecca Golley, Rebecca Byrne, Athira Rohit, Therese Kearns, Sarah Hanieh, Beverley-Ann Biggs, Julie Brimblecombe

**Affiliations:** 1 Nutrition Program, Wellbeing and Preventable Chronic Disease, Menzies School of Health Research, Casuarina 0810, Northern Territory, Australia; dani.kennedy@menzies.edu.au (D.K.); Athira.rohit@menzies.edu.au (A.R.); Julie.brimblecombe@monash.edu.au (J.B.); 2 Nutrition and Dietetics, College of Nursing and Health Sciences, Flinders University, Bedford Park 5042, Australia; rebecca.golley@flinders.edu.au; 3 Institute of Health and Biomedical Innovation, Centre for Children’s Health Research, Queensland University of Technology, Brisbane 4101, Australia; ra.byrne@qut.edu.au; 4 Child Health, Menzies School of Health Research, Casuarina 0810, Northern Territory, Australia; therese.kearns@menzies.edu.au; 5 Department of Medicine at the Peter Doherty Institute for Infection and Immunity, The University of Melbourne, Victoria 3010, Australia; shanieh@unimelb.edu.au (S.H.); babiggs@unimelb.edu.au (B.-A.B.); 6 Nutrition, Dietetics and Food, Faculty of Medicine, Nursing and Health Sciences, Monash University, Victoria 3168, Australia

**Keywords:** diet, questionnaire, Indigenous, public health, food

## Abstract

The Menzies Remote Short-item Dietary Assessment Tool (MRSDAT) can be used to derive a dietary index score, which measures the degree of compliance with the Australian Dietary Guidelines. This study aimed to determine the relative validity of a dietary index score for children aged 6–24 months, living in a Remote Aboriginal Community (RAC), derived using MRSDAT. This validation study compared dietary index scores derived using MRSDAT with those derived from the average of three 24-h recalls. Participants were aged 6–36 months at the first dietary assessment and were living in a RAC. The level of agreement between the two methods was explored using Lin’s concordance correlation coefficient (CCC), Bland-Altman plots, weighted Cohen’s kappa, and Fischer’s exact and paired *t*-tests. Forty participants were recruited. The CCC was poor between methods (*R* = 0.35, 95% CI 0.06, 0.58), with MRSDAT estimating higher dietary intake scores for all food groups except fruit, and higher dietary quality scores by an average of 4.78 points/100. Community-based Aboriginal researchers were central to this validation study. MRSDAT was within the performance range of other short-item dietary assessment tools developed for young children, and shows promise for use with very young children in RACs.

## 1. Introduction

A healthy diet in early childhood is essential to ensure adequate growth and development [1], and lifelong health [2]. While this is generally well known by parents, complex social and food security issues make providing young children with an optimal diet challenging for caregivers in remote Australian Aboriginal Communities (RAC) [3,4]. Food accessibility issues are reported to impact the diets of adults living remotely [5,6],with sales data from community food stores suggesting that diets vary substantially during a pay cycle and that meat and vegetables are in the top four categories of expenditure only in the early income cycle period, not featuring in the low money period [7]. There is limited literature available characterising the diets of very young children living in RACs, particularly around the critical time of solids introduction (around six months of age) [2,3]. The small body of literature that is available highlights that infant and toddler diets in RACs are suboptimal and calls for further research [3,8]. Contributing to this gap in the literature are the complexities faced in collecting dietary intake data and assessing diet quality in a RAC population [3,9].

Dietary assessment presents many challenges in the most ideal of settings [10]. In any research study, a balance must be struck between the accuracy of available methods for dietary assessment and the practicality and feasibility of the dietary assessment process for the study population. While weighed food records may theoretically provide the most valid data, they are time consuming, burdensome for participants, require a high level of literacy and numeracy, and as such are often poorly executed [11]. Twenty-four-hour recalls, while less burdensome for participants, are also resource intensive as they typically require specialist administration and considerable time [11]. Food Frequency Questionnaires (FFQ) are more commonly employed in research, due to their comparatively low cost, ease of administration and low participant burden [10]. The FFQ method when administered verbally is especially appropriate for use with populations in which differences exist in terms of language and literacy between researchers and participants [12].

Another advantage of using FFQ is the potential ease in comparing the whole diet against an established reference of diet quality, including national dietary guidelines [13]. Increasingly, short-form FFQ or surveys are emerging, providing a quick measure of overall diet quality and adequacy [14,15]. The Dietary Guideline Index is a scoring system developed in Australia that enables users to compare dietary intake data derived from a long or short form FFQ [16,17], or 24-h recalls [18,19], against the Australian Dietary Guidelines in both adult (DGI) and child populations (DGI-CA). While highly relevant to diet quality assessment with DGI-CA, the short-form dietary assessment methods developed to date are not developed for use in RACs, and therefore would not capture the unique dietary habits seen in remote community populations. Unless specifically designed for use in the RAC setting, FFQs use inappropriate language, do not incorporate culturally diverse foods and are often too resource intensive or require a level of English literacy that is typically not found in the RAC population.

A recent review of studies that validate FFQs for dietary assessment in children aged 12–36 months identified 17 tools, none of which were developed in Australia nor applicable to RAC populations [13]. Indeed, a review of the literature indicates that there are no short-item tools to assess the diet of very young children that have been validated for use in RACs in Australia [14,15]. As such, a short-item questionnaire has been developed to assess the dietary quality of mothers and their young children (2–4 years) in the RAC setting for the Pregnancy and Adverse Neonatal Diabetes Outcomes in Remote Australia (PANDORA) longitudinal birth cohort study [20]. Briefly, the Menzies Remote Short Item Dietary Assessment Tool (MRSDAT) comprises 30 questions that capture consumption of fruits and vegetables, meat, dairy, breads and cereals, fats, wild hunted and harvested foods, and discretionary foods [21]. In reliability testing with PANDORA participants, the questions and response categories were found to be acceptable and 83% of questions and responses tested were reliable [22]. This short-item dietary questionnaire therefore appears to be an appropriate alternative for complex, resource intensive dietary tools when working with Aboriginal Australians in remote settings, but its ability to accurately estimate diet quality is yet to be assessed.

This study therefore aimed to determine the relative validity of the DGI-CA score for children aged 6–24 months living in a RAC, derived using the MRSDAT.

## 2. Materials and Methods

### 2.1. Study Design

This dietary validation study compared the DGI-CA scores derived using the MRSDAT with those derived from multiple 24-h recalls. While 24-h recalls are known to be vulnerable to recall bias and intentional dietary distortion, they are generally accepted to have greater precision than FFQ [13] and are the most appropriate reference measure in studies with language and literacy issues [13,23].

The data for this validation study were collected from a sub-sample of participants in a larger cross-sectional observational study, hereafter referred to as the growth and nutrition study. The growth and nutrition study aimed to document the health status of Aboriginal children aged 0–24 months living in a RAC. Data measuring height, weight, presence of anaemia, iron deficiency and inflammation, dietary intake, food security status, infection status, gut health, hospitalisations, antibiotic use, iron therapy and maternal antenatal history were collected to provide a comprehensive overview of child health in the first two years of life and explore important determinants of anaemia, infection and child undernutrition in this setting. Written consent from the legal guardian of the child was obtained for all participants. The study was conducted in accordance with the Declaration of Helsinki and ethics approval was provided by the Human Research Ethics Committees of the Northern Territory Department of Health and Menzies School of Health Research (project number 2017–2814), and the Royal Melbourne Hospital.

### 2.2. Participants and Sampling

All children aged 0–24 months (~120) and their parents, living in the community and surrounding Homelands, were eligible to participate in the growth and nutrition study. Children were identified by staff at the local health clinic and playgroup, and from home visits. Aboriginal researchers, comprised of Aboriginal Health Practitioners and Aboriginal Community Based Researchers, explained the research project to mothers/caregivers and obtained written informed consent from those wanting to participate. Note that a number of children turned two during the study and were therefore between 24 and 36 months at dietary data collection. A sample size calculation to determine the minimum number of participants required for the study to have adequate power was not conducted, as these calculations require the expected agreement between methods to be known and, given this study is the first to assess this tool, it was not possible to accurately estimate this. Due to time and resource constraints, 50 of the 70 participants of the growth and nutrition study were approached for participation in this dietary validation.

### 2.3. Data Collection

Dietary assessment was conducted during three two-week data collection visits to the community, occurring in October and November 2017, and January 2018. This corresponds to the build-up and tropical monsoon seasons in the area. The dietary assessment team comprised three local Aboriginal researchers (R.D., V.G., Y.D.), a senior research dietitian (J.B.), a nutrition research assistant (D.K.) and a student dietitian. The dietary assessment team was provided with the contact details of participants, who had agreed to the dietary assessment component of the growth and nutrition study, and contacted participants to organise a time to meet. All dietary assessments were completed at participants’ homes, the local playgroup or an alternative location of their choosing. Sessions were completed in a mix of English and the local language, with the Aboriginal researchers translating questions and responses as needed.

**Figure 1:**
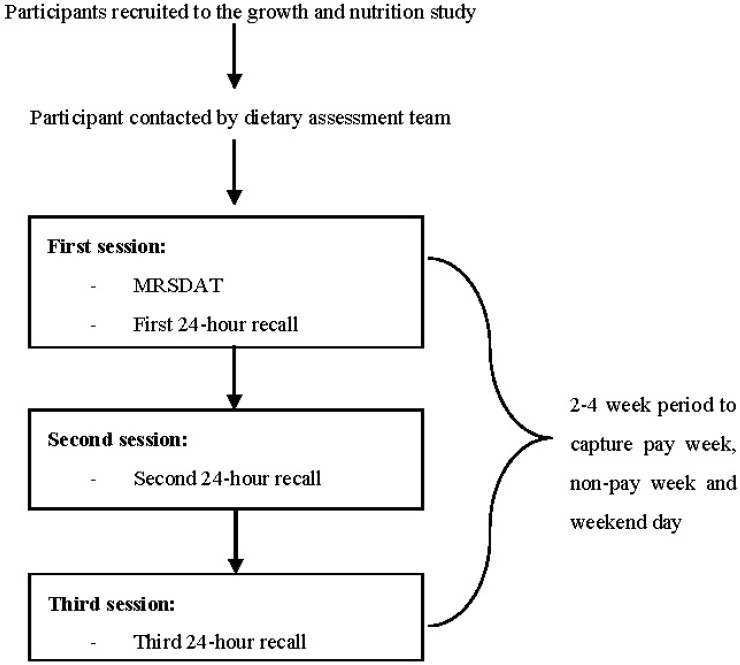
Data collection process and timeline. A Menzies Remote Short item Dietary Assessment Tool (MRSDAT).

Dietary assessment was completed over three sessions (Figure 1). The MRSDAT was the new method and therefore administered first, followed by the first 24-h recall [23]. The second and third 24-h recalls were then conducted over a 2–4-week period to ensure that all three recalls captured a pay week, non-pay week and weekend day [3]. The first dietary assessment for six participants was completed by the senior research dietitian supported by local researchers (V.G. or Y.D.), with all the remaining assessments completed by one researcher (D.K.) supported by one or two of three local researchers (V.G., R.D., and Y.D.). Local researchers translated questions and responses into the local language when needed.

### 2.4. The MRSDAT

A description and reliability assessment of the MRSDAT has previously been reported [22]. Briefly, the MRSDAT is a 30-item diet quality questionnaire developed to assess the dietary quality of mothers and their young children (2–4 years) in the RAC setting. All major food groups are assessed, with six items estimating consumption of fruits and vegetables, three dairy foods, three breads and cereals, six meat and meat alternatives, five healthy fats, five discretionary foods known to have high consumption in RACs, and, finally, two items examining breastfeeding and traditional food consumption. Illustrative food serving images are built into the iSurvey (Harvest Your Data, Wellington, New Zealand) version of the MRSDAT to assist with respondent estimation of serving size and consumption frequency. Images included food type prompts (for example different types of red meat: beef mince, kangaroo steak, canned processed red meat) and serving size prompts (for example an image of a hand with the palm circled, captioned ‘one serve red meat’). The reference period for assessing intakes suggested to participants was the preceding fortnight to capture ‘usual’ short-term intake, which in the participant age group for this study is changing rapidly [13].

The MRSDAT was administered verbally by a member of the research team using an iSurvey version of the tool on an iPad. MRSDAT data were downloaded from the iSurvey website into Microsoft Excel 2010 (Microsoft, Redmond, DC, USA) for cleaning and scoring.

### 2.5. Twenty-Four-Hour Recall Dietary Assessments

The 24-h recall procedure followed a standardised three-pass method [24,25]. The child’s caretaker was asked to recall everything the child ate or drank in the previous 24-h, starting from midnight on the previous day, with quantities estimated using household measures provided by the researchers (metric cups and spoons), with a researcher noting responses by hand on a hardcopy document. The caregiver was specifically asked if anyone else had spent time caring for the child during the relevant 24-h period, including other children. If others did care for the child during this 24-h period, the caregiver was asked if they had provided the child with any food or drink, and details were recorded if they were available. The second and third 24-h recalls were completed following the same process. The 24-h recall data was entered into a custom-made Access database (Microsoft, Redmond, DC, USA) built using the 2011–13 AUSNUT food composition database [26], and the Australian Health Survey discretionary food list and food and supplement classification data cubes [27]. All data were entered by one researcher (D.K.) and checked by a second (E.T.). Data cleaning followed the process outlined by Mauch and Magarey [25].

### 2.6. Analysis

#### 2.6.1. Calculating Dietary Index Scores

Intake data from the MRSDAT and 24-h recalls were converted to daily food group servings using reference servings from the national dietary guidelines [21]. Daily intake data from the three 24-h recalls were then averaged to calculate average daily food group servings for each participant, and these data were checked for plausibility. These data were then used to calculate diet quality scores, derived from the MRSDAT and the averaged 24-h recalls. The diet quality scoring method used was based on the previously-reported DGI-CA for children aged 4–11 years [16,18]. The DGI-CA reflects key elements of the Australian dietary guidelines, comprising nine ‘indicators’: 10 points are allocated for each of the five food groups (fruit, vegetables, meat and alternatives, dairy, breads and cereals); 10 points each for three additional recommendations (dietary variety, healthy fats and water consumption); and 20 points for intake of discretionary foods, for a total of 100 points [16,18].

A number of modifications to the DGI-CA scoring system were necessary due to the younger age of the participants in the current study, and the RAC setting. First, the Australian dietary guidelines [21] were used to modify the DGI-CA to reflect recommended food group servings for children aged 0–3 years. Second, given the small number of servings recommended for children aged 0–3 years, no minimum serving limit was applied when calculating the dietary variety indicator. Third, no estimations of dairy intake were made and the dairy indicator was excluded from the DGI-CA scoring. MRSDAT was initially developed for a non-breastfeeding population (2–4-year olds) and, in predominantly milk-fed infants it is therefore unable to distinguish between different levels of dairy intake, making the dairy indicator less useful for this age group. Finally, the indicator for water consumption was modified to focus on sugar sweetened beverage (SSB) consumption instead, as store sales and national nutrition data indicate this to be a particular issue in the RAC setting [5,28]. Maximum scores were given for intakes that fully met the recommended servings or guideline, and minimum scores for zero intakes or complete non-compliance with recommendations. In-between intakes were awarded a proportion of the possible score. Total DGI-CA scores were calculated by summing all indicator scores, with a maximum possible score of 90. This was scaled to 100 to enable comparisons with other studies, acknowledging the modifications above.

#### 2.6.2. Statistical Analyses

Descriptive statistics (mean/median and measures of dispersion) were calculated for total DGI-CA scores, scores for each individual DGI-CA indicator and food group intakes for both the MRSDAT and averaged 24-h recalls. Relative validity testing involved a range of approaches. The level of agreement between the derived DGI-CA scores was compared using Lin’s concordance correlation coefficient (CCC) [29,30] as it accounts for both the linear relationship between and the slope of the line relating the two sets of scores and is thought to be robust with as few as 10 data pairs [30]. Bland-Altman plots were used to graphically display agreement between methods, with mean difference, limits of agreement (LOA) and proportional bias assessment calculated using linear regression [23,29]. The ability of the MRSDAT to accurately rank participants within DGI-CA quartiles was assessed using weighted Cohen’s kappa [23]. Paired samples *t*-tests, Wilcoxon signed-rank, Fisher’s exact and Bland-Altman plots were also used in secondary analyses of individual indicators and food groups to explore the overall results. Data for all participants regardless of the number of 24-h recalls completed were analysed in the overall analyses, with secondary analyses exploring data from only those participants who completed all three 24-h recalls. IBM SPSS Statistics version 24 (IBM Corp, Armonk, NY, USA) was used for statistical analyses.

## 3. Results

Forty participants were recruited, with the majority of nutritional data collected between the age of 1–2 years (55%) (Table 1). A high proportion of children were being breast fed (*n* = 34, 85%). Of the 40 participants all completed the first dietary session (MRSDAT and first 24-h recall), 36 (90%) completed the second, and 33 (83%) completed all three dietary assessment sessions. The time of follow up ranged from 6–30 days, with a median of 16 days between the 1st and 3rd recalls. The participant characteristics are presented in Table 1. Parents reported that their child ate less than usual at 21 of the 24-h recall sessions (20%), more than usual at 22 (20%), and their usual intake at 65 (60%) sessions. A small number of children were consuming dietary supplements (iron 4, (10%) and vitamin C 2, (5%)).

### 3.1. Relative Validity of Daily Food Group Intakes

Table 2 reports participant daily food group intakes derived from the two methods. Relative to the 24-h recalls, the MRSDAT had higher estimates across all food groups, except fruit (Table 2). While the median reported intakes for vegetables differed by only 0.04 servings between the two methods, and breads and cereals differed by 1.19 servings per day, Wilcoxon signed-rank test only showed the meat and vegetable intakes to be significantly different (*p* < 0.001 and *p* = 0.04, respectively).

### 3.2. Relative Validity of DGI-CA Indicator Scores

**Figure 2:**
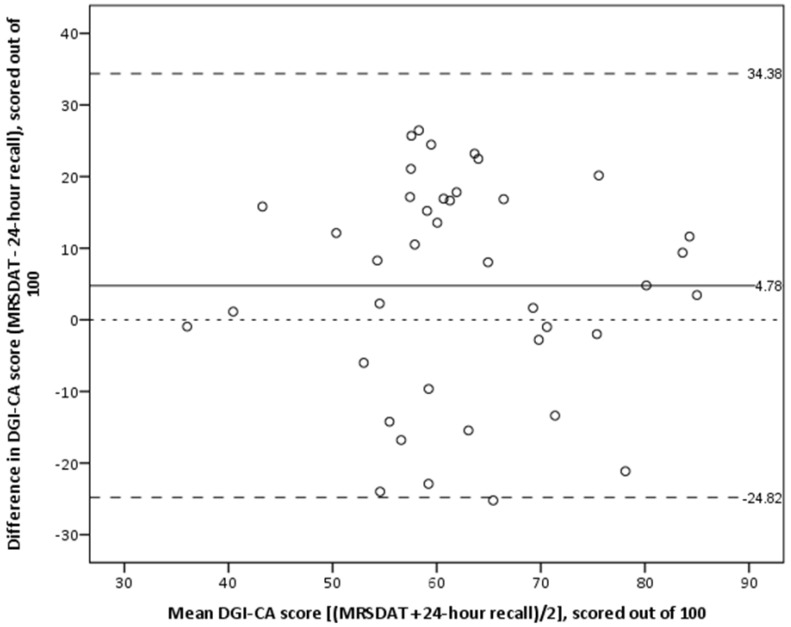
Bland-Altman plot showing agreement between total Dietary Guideline Index—Children and Adolescents (DGI-CA) scores, derived from the Menzies Remote Short-item Dietary Assessment Tool and 24-h recalls (scaled to be out of 100). Mean difference (bias) is represented by the solid line, the upper and lower limits of agreement by the longer broken lines, and the smallest broken line represents 0.

The Lin’s CCC for the total DGI-CA scores, derived from MRSDAT, compared with the averaged 24-h recalls was 0.35 (95% CI 0.06, 0.58). Although there are no formally-defined agreement ranges, this result would generally be considered to reflect poor agreement. Paired samples *t*-test indicated the MRSDAT tended to estimate higher DGI-CA scores by an average of 4.3 points when scored out of 90, and 4.78 points when scored out of 100. The Bland-Altman graph presented in Figure 2 demonstrates that although this bias is small, the LOA are wide (Upper LOA = 34.38, Lower LOA = −24.82 when scored out of 100). This suggests that the small bias reflects that the MRSDAT-estimated DGI-CA scores were both higher and lower to a similar degree compared with those derived from 24-h recalls. Consistent with this, regression showed there to be no proportional bias (slope = −0.05, *p* = 0.83). Secondary analyses showed that the MRSDAT-estimated DGI-CA scores were higher compared with 24-h recalls for all participants <12 months (*n* = 12, 100%), while they were lower for a greater-than-expected proportion of participants aged 1–2 years and 2–3 years (*n* = 10, 46% and *n* = 4, 67% respectively). This was statistically significant (*p* = 0.004). Excluding participants <12 months from the overall analysis resulted in a smaller overall bias (mean difference in DGI-CA scores 1.60 points/100), however the wide LOA persisted (Upper LOA = 34.13, Lower LOA = −30.90/100).

Secondary analyses of individual dietary indicators showed significantly higher scores for meat and wholegrain indicators, and significantly lower dietary variety scores, when estimated by the MRSDAT compared with scores derived from 24-h recalls (all *p* < 0.001, Table 3). For the meat indicator score, this bias was proportional; with the increasing indicator score, the difference between the MRSDAT and 24-h recalls scores was reduced (slope −0.79, *p* < 0.001). The regression for the wholegrain indicator showed a borderline-significant proportional bias in the opposite direction (slope 0.48, *p* = 0.05) and this was also the case for the breads and cereals indicator (slope 0.5, *p* = 0.03); with increasing indicator scores, the difference between MRSDAT and 24-h recall derived scores increased. For all other indicators, secondary testing identified no statistically significant differences in indicator scores, despite the MRSDAT-derived scores tending to be higher for all indicators excepting dietary variety and discretionary foods, with no proportional bias in their estimation (Table 3). Given that the discretionary indicator is negatively scored (higher intakes of discretionary foods resulting in lower scores), lower MRSDAT discretionary indicator scores are consistent with the MRSDAT, tending to estimate higher intakes of all foods.

Two scoring systems were proposed for calculating the dietary variety score for the MRSDAT: system A distributed the overall 10 points across different foods within each food group (for example, distributing 2 points overall between intakes of red meat (0.5 points), white meat (0.5 points), fish (0.5 points) and eggs (0.5 points)), enhancing spread; and system B awarded points for overall consumption of any food within a food group (for example, 2 points for any meat consumption). System B is more similar to that used to score the 24-h recall data. The comparison between MRSDAT System A and 24-h recall estimates of dietary variety is presented in Table 3 and shows them to be significantly different. When system B was used to score the MRSDAT data the difference was attenuated (MRSDAT median 10.0, IQR 10.0–10.0, *Z* = −1.73, *p* = 0.08), however using system B makes little difference to the overall results (Lin’s CCC R = 0.35 (95% CI 0.09, 0.57)), mean difference 7.41 points/100, LOA −20.95, 35.77).

### 3.3. Percentage Agreement between Methods

The MRSDAT includes two questions that do not quantify intake, these assessing whether a child is breastfed and consumes wholegrain bread. Kappa showed there was moderate agreement between methods for determining whether a child is still breastfed (0.41, *p* < 0.001), with only one child in 40 being differently classified between the methods (MRSDAT Yes = 33, No = 7; 24-h recalls Yes = 34, No = 6; 97.5% agreement). However, there was only slight agreement between the methods when determining consumption of wholegrain bread (0.09, *p* = 0.17) [31].

Ranking comparisons showed that 35% of individuals were placed in the same quartile of DGI-CA score using the different methods, representing only slight agreement (*k* = 0.16, *p* = 0.16). When considering only those participants who completed all three 24-h recalls, this increased the percentage of individuals placed in the same quartile to 40%, representing a fair agreement *k* = 0.20, *p* = 0.09 [31].

## 4. Discussion

The MRSDAT is the first validated short-item dietary assessment tool developed specifically to assess the diets of very young (<4 years) Indigenous Australians and their parents in remote settings [22]. The development of the MRSDAT also addresses the recent call for short dietary assessment tools for children aged <2 years, suitable for dietary index applications [15]. Determining the relative validity of this tool is essential for interpreting results of future epidemiological and intervention studies in which it may be used to assess diet [23]. In this preliminary relative validity study, the MRSDAT consistently estimated higher daily food group intakes and DGI-CA scores compared with multiple 24-h recalls. Food group intakes and indicator scores with the furthest agreement were meat, breads and cereals, and fats, with the point estimates for fruit and vegetables demonstrating the closest agreement.

These results are broadly consistent with dietary validation literature. Given the well-known difficulties in assessing habitual (usual) dietary intake, in considering the results it is important to note that the reference method is not necessarily a more accurate measure of intake [11,32]. Underreporting of intake is regularly documented with 24-h recall methods [23,24,32,33,34], thus the higher dietary intake estimates from the MRSDAT are not surprising. Indeed, two reviews of short-item dietary assessment tools, used to assess the diets of children and adolescents, demonstrate that FFQs consistently overestimate compared with reference methods, to a similar degree of bias as that shown here [14,15]. With regard to ranking agreements, the results of the present study are within the range of those from a study in older rural/remote Australian children [35] and strikingly similar to those with participants in the birth to preschool-age group [36,37,38,39,40]. The MRSDATs performance when estimating DGI-CA was also comparable to that demonstrated by the Short Food Survey, which scored the DGI-CA 16 points higher on average in a group of 4–11-year-olds compared with 24-h recalls [16]. While these results are not directly comparable due to the modifications made to the DGI-CA scoring system for this study, overall, they suggest that the performance of the MRSADT falls within the range of other short-item FFQs.

A number of practical factors may have also contributed to the higher daily food group intake estimates from the MRSDAT found in this preliminary study. A monsoonal trough hit the study region at the beginning of the third data collection round, which resulted in extensive power outages that prevented families from purchasing food. Compounded by the food security issues already present in the community, this was reflected in the 24-h recalls on the subsequent day of simply recording breastfeeds, with little to no food at all consumed by children. Further, a small number of children were unwell during the study period and therefore had reduced intake, thus illness in these age groups accompanied by the small volumes of food eaten accords less opportunity for within-day dietary variety and intake volumes. This is reflected in the number of parents reporting that their children ate less than usual, at 20% of 24-h recall sessions. While there were equal parental reports of more-than-usual child intakes (20%), it is unlikely that the degree to which children ate more than usual was comparable to the impact of having no food intake at all in the instances when they were reported to eat less than usual. In addition, the dietary assessment team, in particular the local researchers, noted that they felt there was underreporting of less-than-usual consumption due to parents feeling shame about difficulties in providing food for children on occasions. These issues, in sum, highlight the strength of the FFQ and a limitation of the multiple 24-h recall method for assessing ‘usual’ intake, particularly in populations that experience volatile food access, such as RACs. On the other hand, the FFQ is not necessarily designed to capture the extent of food insecurity.

Despite dedicated personnel staying in community for extended periods during data collection, only 50 participants were able to be approached for participation in this study, with 80% participating. It is common for families to move between community and the wider homelands, and between houses within community, which made locating participants from the growth and nutrition study to request their participation in the dietary validation challenging. Likewise, ceremonial business associated with the funeral of a notable community member meant it was not appropriate to visit families’ homes for a time, limiting the number of participants approached. Further, social issues affecting some families within the community impacted parents’ availability to participate. Finally, the dietary assessment team also thought that the social impacts of historical policies around child protection impacted willingness to participate. Nonetheless, an 83% full protocol completion rate for this sub-study, achieved over a spread of days for each participant (pay week, non-pay week and weekend day), despite the challenging conditions outlined, provides confidence in the recommendations made from these results. Additionally, guidelines outlining minimum sample size requirements for dietary validation studies are not available, and the results presented are descriptively interpreted; it is unlikely that increasing the sample size of this study would change either the point estimates or the interpreted agreement between methods in a meaningful way.

The intensive involvement of local community researchers further strengthens confidence in the accuracy of the data collected and enabled community capacity building, increasing awareness of key dietary habits important for good health within the community. Not only were community researchers instrumental in achieving the high completion rate, but the assessments being led or supported by community researchers allowed participants to speak in their own language and use common terms to estimate amounts consumed. A further strength of this study is the robust analysis, using appropriate methods for assessing agreement, and specifically the use of CCC, which is appropriate for use with samples as small as 10. Although the lack of use of Pearson’s correlation limits our ability to directly compare with other similar studies, the use of correlation is incorrect and inappropriate to assess agreement, and our statistical methods therefore represent the most robust methods available [29].

A number of suggestions can be made for adaptions to the MRSDAT and the DGI-CA scoring system to improve outcomes in future validity testing. In the population of the current study there is typically an overreliance on breastfeeding and, as such, growth faltering impacted by suboptimal solids introduction. Therefore, excluding the dairy indicator is less problematic than in other population groups. However, it may be of benefit to modify the breastfeeding question responses to include ‘breastfeeding’, ‘formula feeding’ and ‘no breastfeeding or formula feeding’. There is also the potential to adapt the DGI-CA scoring relating to wholegrain consumption to extend beyond bread to other forms of wholegrain, such as brown rice, to enable greater demonstration of this guideline in 24-h recalls. These adaptions are more relevant to younger populations with limited meal volumes and would not be as necessary with adult populations. In terms of using any tool in the RAC population, due to the fundamental differences in how time is conceptualised, demarcated and responded to, defining the time period of dietary assessment is difficult. Here it was found that using the pay cycle (that is, ‘this pay’) as a period to define the dietary assessment window was useful. It should also be noted that the MRSDAT aims to capture usual intake over the preceding fortnight (retrospective intake), while the 24-h recalls were completed such that they captured intake of the 2–4 weeks after the MRSDAT assessment (prospective intake). While this limits the ability of the tools to estimate the same window of dietary intake, completing the 24-h recalls first and the MRSDAT last risks having no MRSDAT assessment if participants are unable to be followed up. This limitation would also result in higher 24-h recall derived intakes, which is not the case in the present study.

Future studies to determine the consistency and responsiveness of the MRSDAT are necessary. In particular, the MRSDAT may perform better in older age groups with established solids consumption. Additionally worth noting is that the MRSDAT was not self-administered in this study, but verbally administered by trained community researchers with pictorial aids through iSurvey. This was necessary given previous reports indicating that self-administered FFQs are not feasible in the RAC population [41], however research into the performance of the MRSDAT when self-administered would be of value.

## 5. Conclusions

The performance of the MRSADT for estimating daily food group intakes and DGI-CA in this preliminary validation study suggests it could be improved, however the MRSDAT was within the performance range of other FFQs developed for young children and is the first validated tool appropriate for use with very young children in RACs. A number of recommendations have been made to enhance performance in future validity testing. Additionally, this research highlights the importance of drawing on the skills and expertise of local researchers in RACs.

## Figures and Tables

**Table 1:** Participant characteristics.

Participant Characteristic		*n* (%)
Gender		
	Female	20 (50)
	Male	20 (50)
Age at first session		
	<12 months	12 (30)
	1–2 years	22 (55)
	>2 years	6 (15)
Use of dietary supplements		
	Yes	6 (15)
	No	34 (85)
Currently breastfeeding		
	<12 months	11 (92)
	1–2 years	19 (86)
	>2 years	4 (67)

**Table 2:** Comparison of the median daily food group intakes estimated from the MRSDAT ^a^ and the 24-h recalls. Caregiver reported intake for 40 children aged 6–36 months from a remote Aboriginal community in Northern Australia.

Food Group	MRSDAT, Median Serves per Day (IQR)	Average of 24-h Recalls, Median Serves per Day (IQR)	*Z* Statistic, *p*-Value ^b^
Vegetables	0.67 (0.42, 1.23)	0.63 (0.12, 0.97)	−2.07, *p* = 0.04 ^c^
Fruit	0.42 (0.14, 1.75)	0.77 (0, 1.35)	−0.49, *p* = 0.63
Breads and cereals	3.00 (0.57, 3.00)	1.81 (1.53, 2.87)	−0.80, *p* = 0.43
Meat	1.81 (1.36, 2.31)	0.70 (0.35, 1.14)	−4.37, *p* < 0.001 ^c^
Sugar sweetened beverages	0 (0, 0.05)	0 (0, 0)	−1.02, *p* = 0.31
Discretionary foods	0.28 (0, 0.70)	0.16 (0, 0.46)	−1.42, *p* = 0.16

^a^ Menzies Remote Short Item Dietary Assessment Tool (MRSDAT); ^b^ Wilcoxon signed-rank test used, as all data was not normally distributed. ^c^ Statistically significant results.

**Table 3:** Relative validity of the Dietary Guideline Index—Children and Adolescents (DGI-CA) and indicator scores, derived using MRSDAT.

Indicator (/Possible Score)	MRSDAT Mean, SD,	Average of 24-h Recalls Mean, SD, Median (IQR) or *n* (%)	*t*-Test or Wilcoxon Signed-Rank Test Result	Slope of Bias	*p*-Value for Slope of Bias
Total DGI-CA score /90	58.37, +/−11.92	54.07, 12.32	4.30 (−0.05, 8.66) ^a^	−0.05	0.83
Total DGI-CA score /100	64.86, +/−13.25	60.08, 13.68	4.78 (−0.06, 9.63) ^a^	−0.05	0.83
Food groups	median (IQR)				
Vegetables (/10)	5.08 (2.8, 9.95)	3.87 (1.57, 7.72)	−1.70, *p* = 0.09 ^b^	−0.08	0.68
Fruit (/10)	10.00 (4.2, 10.00)	10.00 (0.13, 10.00)	−1.50, *p* = 0.14 ^b^	−0.42	0.16
Breads and Cereals (/5)	3.75 (0.94, 3.75)	2.52 (2.05, 3.79)	−0.25, *p* = 0.80 ^b^	0.52	0.03 ^c^
Meat (/10)	10.00 (10.00, 10.00)	8.27 (4.93, 10.00)	−3.92, *p* < 0.001 ^b, c^	−0.79	<0.001 ^c^
Sugar sweetened beverages (/10)	10.00 (9.47, 10.00)	10.00 (10.00, 10.00)	−1.30, *p* = 0.19 ^b^	0.23	0.41
Discretionary foods (/20)	5.20 (0, 20.00)	13.56 (0, 20.00)	−1.67, *p* = 0.09 ^b^	−0.08	0.72
Food choices	median (IQR)				
Dietary variety, System A (/10)	7.2 (6.27, 8.66)	10.00, (8.00, 10.00)	−4.72, *p* < 0.001 ^b,c^	0.18	0.43
Healthy fats (/10)	6.14, +/−1.04	2.95, 1.42	3.19 (2.60, 3.78) ^a^	−0.68	0.06
Whole grains (/5)	2.5 (0, 5.00)	0 (0, 0)	−4.31, *p* < 0.001 ^b, c^	0.48	0.05 ^c^
Whole grains					
Yes	*n* = 28 (70%)	4 (10)			
No	*n* = 12 (30%)	36 (90)			

^a^ Paired samples *t*-test, reported as mean difference (95% CI); ^b^ Wilcoxon signed-rank test, reported as *Z*-statistic, *p*-value. ^c^ Statistically-significant results.
